# Patient of Congenital Absence of a Lumbar Pedicle With Nerve Root Anomaly Presenting With Ipsilateral Foraminal Stenosis by Vertebral Fracture

**DOI:** 10.1155/2024/2671270

**Published:** 2024-07-30

**Authors:** Shotaro Fukada, Takeru Tsujimoto, Masahiro Kanayama, Fumihiro Oha, Yukitoshi Shimamura, Yuichi Hasegawa, Shogo Fukase, Tomoyuki Hashimoto, Norimasa Iwasaki

**Affiliations:** ^1^ Spine Center Hakodate Central General Hospital, Hon-cho 33-2, Hakodate, Hokkaido 040-8585, Japan; ^2^ Department of Orthopaedic Surgery Hokkaido University Graduate School of Medicine, Kita-15 Nishi-7, Kita-ku, Sapporo, Hokkaido 060-8638, Japan

**Keywords:** congenital absence, lumbar fusion, lumbar pedicle, nerve root anomaly

## Abstract

**Background:** Patients with congenital absence of a lumbar pedicle and nerve root anomaly presenting with ipsilateral foraminal stenosis are extremely rare.

**Case Presentation:** An 80-year-old man had low back and right thigh pain. Radiographs and computed tomography (CT) showed L3 vertebral body fracture and the absence of the right L3 lumbar pedicle. He was diagnosed with L2–L3 right foraminal stenosis caused by an L3 vertebral fracture and underwent lumbar fusion at L2–L3 and L3–L4. Intraoperatively, we confirmed that an anomalous nerve root was divided from the right L2 nerve root near the dorsal root ganglion (DRG).

**Conclusions:** Patients with congenital absence of a lumbar pedicle are less prone to ipsilateral foraminal stenosis because they theoretically have a large space in the foramen. This rare case was caused because of additional instability due to vertebral fracture under the condition of a nerve root anomaly and lumbar degeneration.

## 1. Introduction

Congenital absence of a lumbar pedicle is a rare congenital anomaly with a few reported cases, which was first reported by Norman and Johnson in 1973 [1]. Most reports of aplasia of a lumbar pedicle include asymptomatic incidental diagnoses on diagnostic imaging, back pain, disc herniation, or radiculopathy with degeneration [1–7]. However, patients with congenital absence of a lumbar pedicle and nerve root anomaly presenting with ipsilateral foraminal stenosis are extremely rare. Here, we report the first case of a patient with congenital absence of a lumbar pedicle and nerve root anomaly who underwent lumbar fusion for ipsilateral foraminal stenosis.

## 2. Case Presentation

**Figure 1:**
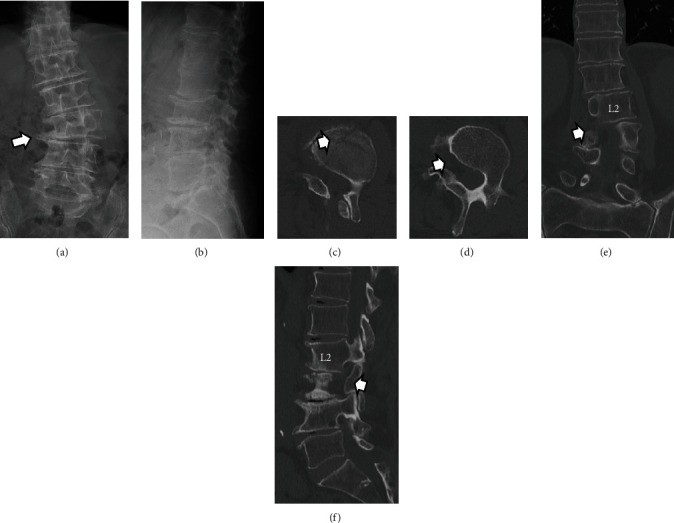
(a, b) Preoperative radiograph and (c–f) computed tomography images of the lumbar spine. (a) White arrows show indistinct right L3 lumbar pedicle, (c) the fracture lines of the L3 vertebra, and (d–f) absence of the right L3 pedicle.

**Figure 2:**
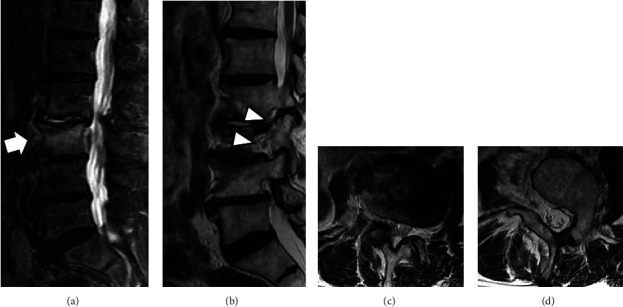
Preoperative magnetic resonance image. (a) STIR midsagittal, (b) T2-weighted right parasagittal, and T2-weighted axial ((c) L2-L3 disc level; (d) L3 pedicle level). White arrow: intensity change due to a L3 fracture. Arrowheads: right L2 (upper) and L3 (lower) nerve root.

An 80-year-old man had a 1-month history of low back and right buttock pain that radiated to the front of his right thigh. Symptoms began upon awakening, and there was no obvious trauma mechanism. His medical history included retinitis pigmentosa and cataracts. One month of treatment with drugs and physical therapy was unsuccessful, and the patient continued to have difficulty walking. The patient was then referred to our orthopedic department. The results of the physical examination, including the results of the motor and sensory tests, were within normal ranges. Lumbar spine radiographs showed degenerative lumbar scoliosis and an indistinct right L3 lumbar pedicle (Figures 1(a) and 1(b)). Lumbar lordosis (LL) was 18°, and L2–L4 Cobb angle was 24°. Computed tomography (CT) revealed a fracture line in the L3 vertebral body and the absence of the right L3 lumbar pedicle (Figures 1(c), 1(d), 1(e), and 1(f)). Magnetic resonance imaging (MRI) of the lumbar spine showed a hyperintense signal in the STIR sequence around the L3 vertebral body and an indistinct L2 nerve root in the right parasagittal view (Figure 2). We could not find any signs of anomalous root by preoperative MRI. The *T*-scores with dual-energy X-ray absorptiometry (DXA) of the femoral neck and lumbar spine were −1.1 and −0.4, respectively. The patient was diagnosed with L2–L3 right foraminal stenosis caused by an L3 vertebral fracture. The Japanese Orthopedic Association (JOA), Roland–Morris disability questionnaire (RDQ), and Oswestry disability index (ODI) were 8/29, 13/24, and 36/100, respectively. Furthermore, visual analog scale (VAS) scores for low back pain (LBP), leg pain, and leg numbness were 70/100, 70/100, and 65/100, respectively. The patient was administered right L2 and L3 nerve root injections, which temporarily resolved the symptoms; however, the thigh pain soon flared up again, and surgery was finally indicated.

**Figure 3:**
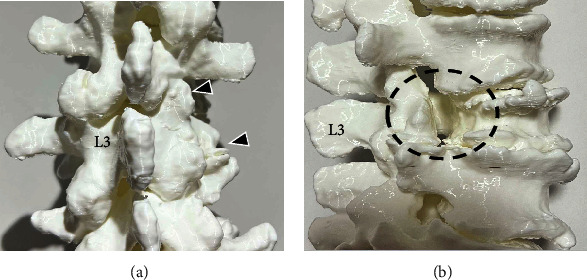
Preoperative computed tomography–based 3-dimensional bone model. (a) Dorsal and (b) right lateral views. Arrowheads show hypoplasia of the right L2 lower articular process (lower) and the right L3 transverse process. Black dotted line shows absence of the right L3 pedicle.

**Figure 4:**
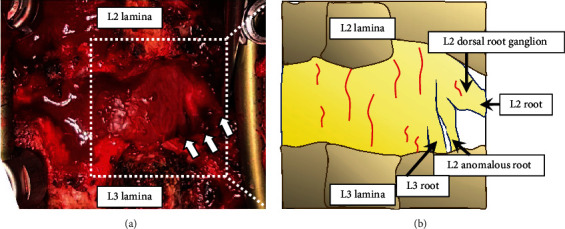
(a) Intraoperative photograph and (b) schema. Three nerve roots occupied the space of the missing right L3 pedicle.

**Figure 5:**
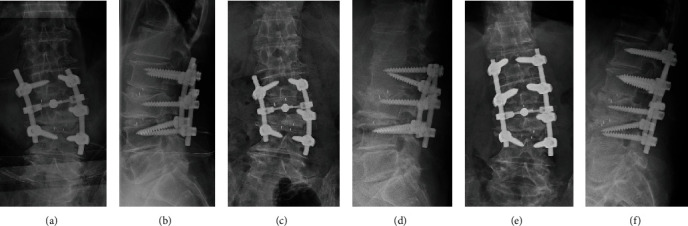
Radiographs at (a, b) immediate postoperative period, at (c, d) 4 weeks postoperatively after primary surgery, and (e, f) additional surgery.

Preoperative CT-based 3-dimensional (3D) bone model was prepared to check the anatomy of the patient's lumbar spine. The 3D model showed hypoplasia of the L2 right lower articular process and L3 right transverse process in the absence of the right L3 pedicle (Figure 3). Transforaminal lumbar interbody fusion (TLIF) at L2–L3 and L3–L4 was performed. Pedicle screws were placed with correct positioning in the L2, L3, and L4 vertebrae. The L3 right screw was not inserted because of the absence of a pedicle. The L2 right lower articular process and L3 upper articular process were then removed. We confirmed that the three nerve roots occupied the space of the missing right L3 pedicle (Figure 4). An anomalous nerve root was divided from the right L2 nerve root near the dorsal root ganglion (DRG). Because there was not enough space to insert a suitable polyetheretherketone cage in the right L2–L3 disc space due to the anomalous nerve root, we performed TLIF at the left side of L2–L3 and L3–L4 with an autogenous bone graft. Postoperative LL was 32°, and L2-4 Cobb angle was 18° (Figures 5(a) and 5(b)).

L3–L4 cage subsidence and L2 pedicle screw loosening occurred within 1 month after surgery. The correction loss of LL occurred because of the cage subsidence, resulting in decreased LL from 32° immediate postoperative period to 16° postoperative 1 month (Figures 5(c) and 5(d)). Therefore, an additional surgery was performed 1 month postoperatively. The loosened L2 screws were replaced with larger screws, and bilateral L1 screws were placed as additional anchors (Figures 5(e) and 5(f)). The JOA, RDQ, ODI, and VAS scores for LBP, leg pain, and leg numbness improved to 19/29, 3/24, 36/100, 15/100, 0/100, and 0/100, respectively, 1 month after the additional surgery. The brace was removed after 3 months.

## 3. Discussion

Generally, patients with congenital absence of a lumbar pedicle are less prone to ipsilateral foraminal stenosis because they theoretically have a large space in the foramen due to the absence of the pedicle. Therefore, patients with a congenital absence of a lumbar pedicle who present with ipsilateral foraminal stenosis are extremely rare. Indeed, previous reports of aplasia of a lumbar pedicle included patients with asymptomatic incidental diagnosis on diagnostic imaging, back pain, disc herniation, or radiculopathy with degeneration [1–7]. To the best of our knowledge, this is the first report of a patient with congenital absence of a lumbar pedicle who underwent lumbar fusion for ipsilateral foraminal stenosis.

This patient had a right L2 nerve root anomaly, resulting in the occupation of the space of the missing right L3 pedicle. Additionally, the L3 vertebra rotated because of degenerative scoliosis. Under this condition, which reduced the intraforaminal space, L3 vertebral fracture caused instability of the lumbar spine. This could be why he developed ipsilateral foraminal stenosis despite the absence of the right L3 pedicle.

Although this patient underwent lumbar fusion at L2–L3 and L3–L4, the bilateral L2 screws had early loosening. This suggests that the proximal anchors were insufficient because of failure to place screws in the L2 right pedicle. In an older patient with congenital absence of a lumbar pedicle and lumbar fracture, multiple anchors may need to be placed even if the loss of bone density is not detected using DXA.

The association between abnormal lumbosacral morphology and nerve root anomalies has long been known [8, 9]. The prevalence of nerve root anomalies has been reported to range from 0.25% to 17.3% [9–12]. Artico et al. reported that 0.25% of the 1200 patients, who underwent CT or MRI, had nerve root anomalies [11]. Kadish and Simmons investigated 100 cadavers and reported nerve root anomalies in 14 lumbosacral vertebrae (14%), most of which were found at the L5–S1 level (52.2%) [10]. Furthermore, they were classified into four categories of nerve root anomalies (Table 1). The patient in this case had a right L2 nerve root anomaly that was divided from the right L2 nerve root near the DRG at the extradural site. Therefore, the patient in this case had a Type IV anomaly.

In conclusion, we reported an extremely rare case of congenital absence of a lumbar pedicle with a nerve root anomaly presenting with ipsilateral foraminal stenosis. This rare case was caused by additional instability due to vertebral fracture under the condition of a nerve root anomaly and lumbar degeneration. If surgeons plan to perform fusion surgery in older patients with congenital absence of a lumbar pedicle, they should be aware of the existence of nerve root anomalies and need for multiple anchors for fixation.

## Figures and Tables

**Table 1:** Classification of lumbosacral nerve root anomalies by Kadish and Simmons [10].

**Anomaly type**	**Morphology**
Type I	Intradural anastomosis between rootlets at different levels
Type II	Anomalous origin of nerve roots
a	Cranial origin
b	Caudal origin
c	Closely adjacent nerve roots
d	Conjoined nerve roots
Type III	Extradural anastomosis between nerve roots
Type IV	Extradural division of the nerve roots

## Data Availability

The data that support the findings of this study are available from the corresponding author upon reasonable request.
